# Hsp70-2 gene polymorphism: susceptibility implication in Tunisian patients with coronary artery disease

**DOI:** 10.1186/1746-1596-7-88

**Published:** 2012-07-26

**Authors:** Mohamed Yahia Hrira, Latifa Chkioua, Afef Slimani, Henda Chahed, Habib Mosbah, Hamda Ben Khaldoun, Salima Ferchichi, Faouzi Addad, Abdelhedi Miled

**Affiliations:** 1 Laboratory of Biochemistry CHU Hached, Sousse, Tunisia; 2 Research Unit 07/UR/06, Faculty of Pharmacy, Monastir, Tunisia; 3 Unit of Research, Genetic and Biologic Factors of Atherosclerosis, Faculty of Medicine, Monastir, Tunisia; 4 Laboratory of Biochemistry and Enzyme Engineering of Lipases, ENIS Sfax, Tunisia; 5 Department of Cardiology, CHU Fattouma Bourguiba, Monastir, Tunisia

**Keywords:** Coronary artery disease, Hsp70-2 genes, Polymorphism, Tunisian patients

## Abstract

**Abstract:**

Coronary artery disease (CAD) is a multifactorial disease where genetic and environmental factors interact in complex ways to cause the disease. Heat shock protein genes are involved in the progress of CAD. This implies that genetic variants of Hsp70–2 genes might contribute to the development of the CAD.

**Aim of study:**

The aim of this study was to characterize statistical correlation of linkage between lipid profiles, polymorphism PstI site of Hsp70–2 gene and CAD.

**Patients and methods:**

This study was carried out on Tunisian patients with CAD recruited from Hospital of Fattouma Bourguiba of Monastir-Tunisia. Polymerase chain reaction and restriction enzymes were used to determine the genotypic distributions in 252 unrelated patients and 151 healthy control subjects. Further, ApoA-I and ApoB as well as the serum total of cholesterol, HDL, triglyceride, and hs-CRP levels were measured.

**Results:**

We showed a decreased level of ApoA-I, whereas the levels of each of ApoB and hs-CRP were increased in patients with CAD compared with control group. In addition our studies of a polymorphic PstI site of Hsp70-2 gene at position 1267 of the Hsp70–2 gene have revealed that the allelic frequency of P2 was significantly more frequent in CAD patients than controls group (p=0.007, OR=1.495). The genotypic distribution showed a high incidence of P2/P2 genotype in CAD patients (0.190) compared to healthy control (0.009) with reach significant difference (p=0.006). The P2 carriers showed a significantly increased of Total-Cholesterol (CT) and C-reactive protein (hs-CRP) levels in CAD patients (p=0.008 and p=0.018, respectively).

**Conclusion:**

The high incidence of P2-Hsp70-2 genotype in CAD patients and the significantly association of P2/P2 genotype with elevated Total Cholesterol and hs-CRP levels, supported that P2–Hsp70–2 genotype has susceptibility implication in CAD and could increased the risk of CAD in Tunisian population.

**Virtual slides:**

The virtual slide(s) for this article can be found here: http://www.diagnosticpathology.diagnomx.eu/vs/1118340895703689

## Introduction

Coronary artery disease (CAD) is a multifactorial disease that combines genetic and environmental factors [1]. CAD is a complex disease with high morbidity and mortality in Tunisian population [2]. There are several emerging risk factors for CAD including markers of oxidative stress, inflammation and autoimmunity. Atherogenesis is a progressive, multi-step process requiring an ordered sequence of events, in which progress along the disease pathway is driven by risk factors [3]. A cell stress response appears to be a pivotal early event in atherogenesis. The heat shock proteins (Hsps) are potential targets for immune responses, as they may be themselves altered during exposure to the stress response, and are usually not found in the extracellular milieu; they may therefore directly contribute to the inflammatory process [4].

Heat shock proteins (HSPs) are a family of proteins that can be expressed in response to a variety of stress stimuli, including reactive oxygen species, toxic metals, inflammatory cytokines, amino acid analogues, oxidative stress, or ischemia. The HSPs play an important role in the maintenance of cellular integrity and viability [5]. HSPs are classified to seven families according to their molecular weight: HSP10, small HSPs (12 – 43 kDa), HSP40, HSP60, HSP70, HSP90 and large HSPs (100 – 110 kDa). HSPs belong to family of stress proteins, which also consist of glucose-regulated proteins (grps), ubiquitin and the lectin chaperones calnexin and calreticulin [6]. Interestedly the 70–kDa family (Hsp70) and according to to the new nomenclature the human genome encodes 13 members of the HSPA (HSP70) family. The most studied genes are HSPA1A and HSPA1B, the products of which differ only by two amino acids and which are believed to be fully interchangeable proteins [6]. In advanced atherosclerotic lesions, HSP70 is over expressed in several cell types, including monocytes, macrophages, dendritic cells and smooth muscle cells. In early atherosclerotic lesions, however, only dendritic cells, which are key cells in the immune response, over express HSP70 [7]. An inverse relation between HSP70 and atherosclerosis has been reported by several groups. low serum HSP70 levels have been suggested to predict the development of atherosclerosis [8]. In hypertensive patients, increased concentrations of circulating HSP70 correlated with decreased intima/media thickness [9]. In another study by Zhu et al., high serum levels of HSP70 were found to be associated with a low risk of coronary artery disease [10]. We have reported that plasma HSP70 concentrations were decreased in patients with carotid atherosclerosis relative to control healthy subjects [11]. 

Previous studies suggested that 1267 Hsp70 polymorphism has a contributory marker in different diseases such as the association between obesity and P2-Hsp70-2 homozygote genotype [12]. It was reported that HSPA1B +1267 allele G was associated with higher serum Hsp70 levels in patients with severe chronic heart failure [13]. A strict correlation was found among 1267 HSP70–2 polymorphism, enhanced pro-inflammatory cytokines and cerebral ischaemia in old type 2 diabetic-atherosclerotic patients [14]. Another study suggests that 1267 HSP70–2 polymorphism and zinc deficiency are independently associated with ischaemic cardiomiopathy [15].

These finding prompted us to investigate, in patients and healthy controls, the potential association of the polymorphism of Hsp70–2 gene with risk of CAD. For this purpose, we evaluated the genotypic frequency of 1267 Hsp70 polymorphism in patients with CAD. The relationship among this disease, biochemical parameters and Hsp70 polymorphism were assessed.

## Materials and methods

### Study population

A total of 252 patients, with coronary artery disease (CAD) were recruited through the Cardiovascular Department of FattoumaBourguiba hospital (Monastir, Tunisia) (Additional file 1: Table S1), who underwent their first coronary angiography for evaluation the presence of CAD were assessed and investigated in the Biochemistry Laboratory of University Hospital of FarhatHached Sousse. Patients who were young (<45 years) and old age (>75 years) and had inflammatory diseases, valvular heart disease, cancers or rheumatoid arthritis were excluded. The Control group includes 151 subjects all, free of any history of obesity, hypertension, dyslipidemia, diabetes mellitus, or CAD. All participants were of Tunisian origin and gave their informed consent for this study.

### Biochemical analysis

For each patient, blood samples were drawn following overnight fasting into tubes containing EDTA and plasma was immediately separated by centrifugation. The biochemical analysis confirmed the coronary artery diagnosis for all studied patients. Total cholesterol, HDL, triglyceride (TG), C-reactive protein (hs-CRP), and apolipoproteins serum levels were measured. Total cholesterol, HDL and TG serum concentrations were assayed by enzymatic techniques (Randox-Antrim, UK). LDL cholesterol was calculated by using the Friedewald formula [16].

Serum ApoA-I, ApoB, and hs-CRP were determined using a highly sensitive immunonephelometric assay (BNII - Dade Behring) [17].

### Genetic analysis

Genomic DNA was extracted from peripheral blood leukocytes using a standard phenol/chloroforme procedure [18]. Polymerase chain reaction (PCR) was carried out in 25 μl total volume containing 500 ng genomic DNA, 0.2 mmol/L dNTP, 0.8 pmol of each primer, 1.5 mM MgCl2 and 1 U DNA Taq polymerase.

The coding sequence of the hsp70–2 gene was amplified from genomic DNA by using sequence specific oligonucleotide primers: the 5’ primer: 5’-TCCGAAGGACTGAGCTCTTG-3’ was used in combination with the 3’ primer: 5’-CAGCAAAGTCCTTGAGTCCC-3’. Amplification was accomplished by initial incubation at 94°C for 5 minutes followed by 30 cycles of incubation at 94°C, 60°C for 1 minute each, 72°C for 3 minutes, followed by a final incubation at 72°C for 10 minutes.

The corresponding PCR products were digested with *Pst*I to assess the polymorphism of the Hsp70–2 at position 1267. The lack of the polymorphism PstI site within the HSP70–2 gene generated a single product of 2075 pb (HSP-P1 allele). The presence of a PstI site was indicated by the cleavage of the 2075bp amplified product to yield fragments of 1139 and 936 bp (HSP-P2 allele).

### Statistical analyses

Statistical analysis was performed using version 17.0 of the Statistical Package for the Social Sciences: SPSS (SPSS Inc., Chicago, Illinois, USA). Data are presented as mean ± standard deviation for continuous variables, or as medians and interquartile ranges for variables with a skewed distribution. Differences between two groups were evaluated by the unpaired Student t test for continuous variables or the non parametric Mann-Whitney test for discontinuous variables. Qualitative variables were assessed with the Pearson’s χ^2^ test. Allele frequencies, genotype frequencies, odds ratios (ORs) and 95% confidence intervals (CIs) were all estimated by Chi-squared analyses and Hardy–Weinberg equilibrium was calculated. A value of p <0.05 was considered significant.

## Results

### Clinic-biochemical parameters and coronary artery disease

Anthropometric and clinical characteristics (age, gender) of the studied population are summarized in Additional file 1: Table S1. We noted a statistically significant increase of total cholesterol, TG, Apo B and hs-CRP and inversely a significant decrease in ApoA-I, and HDL in all patients compared with controls (Additional file 1: Table S1).

### Polymorphism of the Hsp70-2 and coronary artery disease

The genotypic and allelic frequencies of Hsp70–2 polymorphism in CAD and controls groups are shown in Additional file 2: Table S2. Genotype distribution of Hsp70–2 polymorphism was in Hardy–Weinberg equilibrium. The incidence of P2 allele was more frequent in CAD patients (0.432) than controls group (0.337) with reach significance difference (p=0.007). Similarly, the frequency of the P2/P2 was higher in patients (0.190) than in control group (0.099) (p=0.006). These results indicate that the relative risk of coronary artery associated with the Hsp70–2 gene is confined to P2/P2 homozygote (OR= 2.49 [1.284–4.859]). 

### Correlation between Hsp70-2 polymorphisms and clinic-biochemical parameters

Analysis of the clinic-biochemical characteristics of patients with or without the Hsp70–2–P2/P2 is showed in Additional file 3: Table S3. We showed a strongly association between P2 allele and a higher CT and hs-CRP levels in CAD patients with reach significance difference (p= 0.004 and p=0.026, respectively). But this association was not found in controls groups.

## Discussion

Coronary artery disease is characterized by an inflammatory status and it represents the major cause of death in elderly [14]. In Tunisia, the allele and genotype frequencies of Hsp70-2 were observed in several studies [12,19,20] which evaluated a highly significant association between polymorphisms in Hsp70 gene and studied diseases.

In this study we show for the first time the association between lipid profiles, polymorphism *Pst*I site of Hsp70–2 gene and CAD. Biochemical analysis results showed a decreased level of ApoA- and HDL and inversely an increased level of total cholesterol, triglyceride, Apo B and hs-CRP in patients with CAD compared with control group.

Furthermore, a significant correlation of elevated level of some biochemical parameters with CAD was explained by different mechanisms: Elevated levels of LDL cholesterol are associated with an increased risk of coronary heart disease, stroke, and peripheral artery disease. LDL lipoprotein deposits cholesterol along the inside of artery walls, causing the formation of cholesterol plaque. This accumulation causes thickening of the artery walls and narrowing of the arteries which decreases blood flow through the narrowed area [21].

In addition, elevated levels of C-reactive protein (CRP), a protein that appears in the bloodstream during many inflammatory processes, are associated with acute coronary events. CRP may be used as a predictor of cardiovascular disease based on its correlation with the other known cardiac risk factors and their role in the formation of atherosclerosis [22].

Controversial studies regarding the role of Apo A-I as a risk factor of CAD. Indeed, same studies found a positive correlation but others did not [23].

Additionally, P2-CAD patients displayed increased total serum cholesterol, TG and hs-CRP in comparison to P1-CAD patients (Additional file 3: Table S3), while no differences were observed in serum ApoA-I and HDL between P2/P2 and P1/P1 patients, (Additional file 3: Table S3). Accordingly, patients with P2/P2 genotype are more predisposed to plaque accumulation than those with the P1/P1 genotype and are at risk for possible acute coronary events and the progression of CAD.

However, Hsp70-2 polymorphism does seem to affect hs-CRP (Additional file 3: Table S3). The specific role of Hsp70 within the plaques is still unclear and the data are contradictory. A positively correlation was found between serum HSP70 and asymmetric dimethylarginine (ADMA) with high hs-CRP levels in type 2 diabetes patients suggest that both ADMA and HSP70 have similarly an inhibitory function on nitric oxide synthase (NOS) in inflammatory and oxidative stress situation [24]. In addition molecular analysis of a polymorphic *Pst*I site of Hsp70–2 gene lying in the coding region at position 1267 of the Hsp70-2 gene have revealed that the heterozygous P1/P2 genotype was significantly more frequent in patient group and control patient. The homozygous P2/P2 genotype was significantly more frequent in patient group and significantly less frequent in control group (Additional file 2: Table S2). Our results are consistent with results reported in the literature [23]. Patients with P2 genotype are more predisposed to atheromatous plaque accumulation within the walls of the coronary arteries than those with the P1 genotype and are at risk for possible rupture and start limiting blood flow to the heart muscle [24].

Our report highlighted a direct correlation between P2–Hsp70-2 homozygous, elevated level of LDL cholesterol, hs-CRP and CAD. Therefore, a higher significant association was found in patients group compared with controls. Other studies indicate the association of high Hsp70 levels with low CAD risk. The inverse relation between Hsp70 serum levels and CAD are conjectural [25]. This study suggest that the serum level of HSP70 protein is a potent marker for lowered CAD susceptibility and may be helpful, along with other currently recognized risk factors, in more accurately conveying the overall risk of an individual for CAD [25]. Our data seem to suggest that the presence of 1267 Hsp70–2 polymorphism in patients with CAD play a detrimental role in the accumulation of plaques and the formation of atherosclerosis.

The expression level of Hsp70–2 gene could be among factors affecting the regulation of cholesterol rate or other mechanisms exacerbating this step. It has been shown that Hsp70–2 plays a role in the route of synthesis of cholesterol, folding and subcellular localization of hs-CRP protein. Further studies involving larger patient populations will be required to confirm this hypothesis.

## Conclusions

Our findings suggest that homozygous P2–Hsp70–2 genotype is associated with plasma levels of Total-cholesterol and hs-CRP in a large patient population with CAD. Further investigations and works are needed to confirm these results and to clarify the mechanisms underlying this association.

## Consent

Written informed consent was obtained from the patient for publication of this case report. A copy of the written consent is available for review by the Editor-in-Chief of this journal.

## Abbreviations

CAD: Coronary artery disease; ACE: Angiotensin Converting Enzyme; apo A-I: apolipoprotein A-I; apo B: apolipoprotein B; Hsp: Heat shock proteins; TG: Triglyceride; hs-CRP: High Sensitivity C-Reactive Protein; PCR: Polymerase Chain Reaction; OR: Odds Ratio.

## Competing interests

The authors declare that they have no competing interests.

## Authors’ contributions

MYH and LC: wrote the manuscript. AS and HM participated in data analysis. BKH and FA revised the manuscript. SF and AM revised the manuscript and save final approval of the version to be published. All authors read and approved the final manuscript.

## Supplementary Material

Additional file 1 **Table S1. **Clinical and biological characteristics of CAD patients and healthy controls. Click here for file

Additional file 2 **Table S2. **Hsp70–2 genotype distribution in control subjects and in patients with Coronary artery disease. Click here for file

Additional file 3 **Table S3. **Biochemical characteristics of Clinic-coronary artery disease patients and control groups with or without the Hsp70–2–P2/P2. Click here for file
