# Supplementary material for: Hsp70-2 gene polymorphism: susceptibility implication in Tunisian patients with coronary artery disease
Source: Diagn Pathol. 2012 Jul 26;7:88. doi: 10.1186/1746-1596-7-88 (PMC3558340; doi:10.1186/1746-1596-7-88)
Supplement: Additional file 1 — Table S1. Clinical and biological characteristics of CAD patients and healthy controls. [file 1746-1596-7-88-S1.doc]

Additional file 1:

Table 1: Anthropometric and clinical characteristics of the study population

|  | With P2/P2  N=64 | Without P2/P2  N=339 | P value |
| --- | --- | --- | --- |
| Age (years) | 55.25 ±7.79 | 55.55 ±7.4 | NS |
| Sexe M/F | 40/24 | 227/112 | NS |
| BMI (kg/m2) | 29.93 ±2.95 | 27.7 ±1.76 | NS |
| Apo A-I (g/L) | 1.11± 0.34 | 1.21± 0.29 | NS |
| Apo B (g/L) | 0.90 ±0.42 | 1.01± 0.38 | NS |
| Cholesterol total (mmol/L) | 4.73 ±1.2 | 4.22 ±1.08 | 0.003 |
| HDL-c (mmol/L) | 1.11± 0.31 | 1.15± 0.40 | NS |
| Triglyceride (mmol/L) | 1.76± 0.7 | 1.6 ±0.97 | NS |
| LDL-c (mmol/L) | 2.66 ±1.11 | 2.63± 0.93 | NS |
| hs-CRP (mg/L) | 6.72 ± 2.45 | 3.77 ±1.96 | 0.000 |

Additional file 1Additional file 1: Additional file 1: Additional file 1: Bas du formulaire

*p* : significativité statistique ; NS : non significative
