# Supplementary material for: Hsp70-2 gene polymorphism: susceptibility implication in Tunisian patients with coronary artery disease
Source: Diagn Pathol. 2012 Jul 26;7:88. doi: 10.1186/1746-1596-7-88 (PMC3558340; doi:10.1186/1746-1596-7-88)
Supplement: Additional file 2 — Table S2. Hsp70–2 genotype distribution in control subjects and in patients with Coronary artery disease. [file 1746-1596-7-88-S2.doc]

Additional file 2:

Table 2: Distribution of biochemical parameters of patients and controls

|  | Patients  n=252 | Controls  n=151 | P |
| --- | --- | --- | --- |
| Total cholesterol (mmol/L) | 4.57 ± 1.17 | 3.94 ± 0.93 | <0.001 |
| HDL cholesterol (mmol/L) | 1.08 ±0.23 | 1.23 ± 0.51 | <0.001 |
| LDL cholesterol (mmol/L) | 2.61 ± 1.25 | 2.66 ± 0.69 | NS |
| TG (mmol/L) | 1.83 ± 1.03 | 1.33 ± 0.69 | <0.001 |
| Apo B (g/L) | 1.06 ± 0.37 | 0.93 ± 0.35 | <0.003 |
| Apo A-I (g/L) | 1.06 ± 0.31 | 1.34 ± 0.21 | <0.001 |
| Hs-CRP (mg/L) | 5.96 ± 4.79 | 1.84 ±1.12 | <0.001 |

*p* : significativité statistique ; NS : non significative
