# Supplementary material for: Hsp70-2 gene polymorphism: susceptibility implication in Tunisian patients with coronary artery disease
Source: Diagn Pathol. 2012 Jul 26;7:88. doi: 10.1186/1746-1596-7-88 (PMC3558340; doi:10.1186/1746-1596-7-88)
Supplement: Additional file 3 — Table S3. Biochemical characteristics of Clinic-coronary artery disease patients and control groups with or without the Hsp70–2–P2/P2. [file 1746-1596-7-88-S3.doc]

Additional file 3: Table 3: Hsp70-2 Genotype frequencies in control subjects and in patients with Coronary artery disease

| Genotype |  | Controls  (n = 151) |  |  | Coronary artery disease (n= 252) |  | OR | Confidence  intervalle | P value |
| --- | --- | --- | --- | --- | --- | --- | --- | --- | --- |
|  | n | *f* | % | n | *f* | % |  |  |  |
| Hsp70-2 |  |  |  |  |  |  |  |  |  |
| P1/P1 | 64 | 0.423 | 42.38 | 82 | 0.325 | 32.54 | 1.322 | 0.854-2.049 | *0.210* |
| P1/P2 | 72 | 0.476 | 47.68 | 122 | 0.484 | 48.41 | 1.525 | 1.005-2.313 | 0.046 |
| P2/P2 | 15 | 0.099 | 9.93 | 48 | 0.190 | 19.05 | 2.498 | 1.284-4.859 | *0.006* |

Hsp70-2: heat shock protein 70; f: frequencies; OR: odds ratio; NS: not significant.

The chi-square test with Yates correction was used to determine whether significant differences (P value) were observed when patient group was compared with control subjects.
